# Co-cultivation of murine BMDCs with 67NR mouse mammary carcinoma cells give rise to highly drug resistant cells

**DOI:** 10.1186/1475-2867-11-21

**Published:** 2011-06-28

**Authors:** Christa Nagler, Cornelia Hardt, Kurt S Zänker, Thomas Dittmar

**Affiliations:** 1 Institute of Immunology, Witten/Herdecke University, Stockumer Str. 10, 58448 Witten, Germany; 2 Institute of Immunology, University Hospital Essen, Virchowstr. 179, 45147 Essen, Germany

## Abstract

**Background:**

Tumor tissue resembles chronically inflamed tissue. Since chronic inflammatory conditions are a strong stimulus for bone marrow-derived cells (BMDCs) it can be assumed that recruitment of BMDCs into cancer tissue should be a common phenomenon. Several data have outlined that BMDC can influence tumor growth and metastasis, e.g., by inducing a paracrine acting feedback loop in tumor cells. Likewise, cell fusion and horizontal gene transfer are further mechanisms how BMDCs can trigger tumor progression.

**Results:**

Hygromycin resistant murine 67NR-Hyg mammary carcinoma cells were co-cultivated with puromycin resistant murine BMDCs from Tg(GFPU)5Nagy/J mice. Isolation of hygromycin/puromycin resistant mBMDC/67NR-Hyg cell clones was performed by a dual drug selection procedure. PCR analysis revealed an overlap of parental markers in mBMDC/67NR-Hyg cell clones, suggesting that dual resistant cells originated by cell fusion. By contrast, both STR and SNP data analysis indicated that only parental 67NR-Hyg alleles were found in mBMDC/67NR-Hyg cell clones favoring horizontal gene transfer as the mode of origin. RealTime-PCR-array analysis showed a marked up-regulation of Abcb1a and Abcb1b ABC multidrug transporters in mBMDC/67NR-Hyg clones, which was verified by Western Blot analysis. Moreover, the markedly increased Abcb1a/Abcb1b expression was correlated to an efficient Rhodamine 123 efflux, which was completely inhibited by verapamil, a well-known Abcb1a/Abcb1b inhibitor. Likewise, mBMDCs/67NR-Hyg clones revealed a marked resistance towards chemotherapeutic drugs including 17-DMAG, doxorubicin, etoposide and paclitaxel. In accordance to Rhodamine 123 efflux data, chemotherapeutic drug resistance of mBMDC/67NR-Hyg cells was impaired by verapamil mediated blockage of Abc1a/Abcb1b multidrug transporter function.

**Conclusion:**

Co-cultivation of mBMDCs and mouse 67NR-Hyg mammary carcinoma cells gave rise to highly drug resistant cells. Even though it remains unknown whether mBMDC/67NR-Hyg clones originated by cell fusion or horizontal gene transfer, our data indicate that the exchange of genetic information between two cellular entities is crucial for the origin of highly drug resistant cancer (hybrid) cells, which might be capable to survive chemotherapy.

## Background

It is known for decades that tumor tissue resembles chronically inflamed tissue - a matter why tumors have been referred to as "wounds that do not heal" [1,2]. Since chronic inflammation is a strong stimulus for the recruitment of BMDCs [3-5] it can be concluded that the migration of BMDCs into tumor tissues is a common process.

Several lines of evidence indicated that BMDCs, including macrophages and mesenchymal stem cells (MSCs), can trigger tumor growth and metastasis [6-8]. It is assumed that BMDCs can promote a proglycolytic phenotype in tumor cells, thus giving them a survival advantage in hypoxic and inflammatory conditions [9], or promote tumor cell survival through the activation of the integrin-linked kinase (ILK), thereby activating the prosurvival AKT signaling pathway [10]. Another mechanism has been described by Karnoub and colleagues by demonstrating that breast cancer cells stimulated the de novo secretion of the chemokine CCL5 (also named RANTES) from MSCs, which then acted in a paracrine fashion on the cancer cells to enhance their motility, invasion and metastasis [7].

In addition to these mechanisms, which are based on the intercellular communication between BMDCs and tumor cells, cell fusion and horizontal gene transfer (HGT) have also been associated with BMDC mediated promotion of tumor growth and metastasis. Data of Rizvi et al. indicated that BMDCs can fuse with neoplastic intestinal epithelial cells, thereby giving rise to stable tumor cell/BMDC hybrids [4]. Similar data were recently provided by Powell and colleagues, whereby it was shown that not only macrophages, but also T- and B-cells can fuse with tumor cells [11]. Moreover, gene expression analysis revealed a nuclear reprogramming in hybrid cells [11]. Even though both studies did not investigate whether BMDC/tumor cell hybrids do contribute to tumor progression at all, several data of the past years indicated that cell fusion is a common phenomenon in cancer and that hybrid cells possess novel characteristics, which can be definitely linked to tumor progression (for review see [12,13]). These properties include tissue heterogeneity [14,15], an increased malignancy [16,17], drug resistance [12,16] and an enhanced resistance of tumor cells to apoptosis [18]. Additionally, cell fusion has also been suggested as one mechanism how cancer stem cells might originate [13,19,20].

In contrast to animal studies, here it was shown that BMDC × melanoma cell hybrids, either originated by artificial *in vitro *cell fusion or by spontaneous *in vivo *cell fusion, exhibited a markedly enhanced metastatic capacity [21,22], the available data records for "cell fusion in human tumors and the possible outcome" are controversial. Putative cell fusion events have been reported for renal cell carcinoma [23,24], breast cancer [25,26], rectal cancer [27], and multiple myeloma [28,29]. While data of Shabo and colleagues revealed that expression of the macrophage receptor CD163 on breast cancer and rectal carcinoma cells was generally associated with a poorer outcome of afflicted patients, Larsson et al. reported that syncytin expression constitutes as a positive prognostic factor in breast cancer, possibly due to mediating fusions between breast cancer cells and endothelial cells [30].

HGT has also been suggested as a mechanism how tumor cells could receive foreign DNA concomitant with rendering the cells malignancy (for review see: [31]). Several lines of evidence indicated that HGT of chromosomal DNA between tumor cells and tumor cells or tumor cells and normal cells is efficiently facilitated through the uptake of apoptotic bodies [32]. HGT of tumor DNA to endothelial cells *in vivo *gave rise to endothelial cells maintaining the ability to form functional vessels and concurrently express tumor-encoded and endothelial-specific genes [33]. Likewise, *in vivo *gene transfer between interacting human osteosarcoma cell lines was associated with acquisition of an enhanced metastatic potential [34].

In the present study we investigated the outcome of co-cultivation of murine mammary 67NR-Hyg breast cancer cells and murine BMDCs derived from Tg(GFPU)5Nagy/J mice [35]. Our data show that mBMDC/67NR-Hyg clones possessed a marked up-regulation of Abcb1a/b ATP binding cassette (ABC) multidrug resistance transporters, which was correlated to an enhanced resistance of these cells towards chemotherapeutic drugs.

## Results

### Co-cultivation of mBMDCs and murine 67NR mammary carcinoma cells resulted in hygromycin and puromycin double resistant cells

In the present study we investigated the co-cultivation of mBMDCs and murine mammary carcinoma cells (67NR-Hyg). The 67NR cell line was derived from a mammary tumor, which spontaneously arose in a Balb/cfC3H mouse [36]. It is highly tumorigenic, but not metastatogenic [36,37]. To select for putative hybrid cells, a dual antibiotic-based selection strategy [16,17,38] was chosen. Thereby, mBMDCs exhibited EGFP expression and puromycin resistance [35], whereas murine 67NR-Hyg mammary carcinoma cells were stably transfected with a hygromycin resistance. In total, 9 double resistant clones (entitled as mBMDC/67NR-clone X [X = 1-9]) were isolated and propagated. All clones grew well in selection media. For further analysis clones 1 to 3 have been chosen randomly.

**Figure 1:**
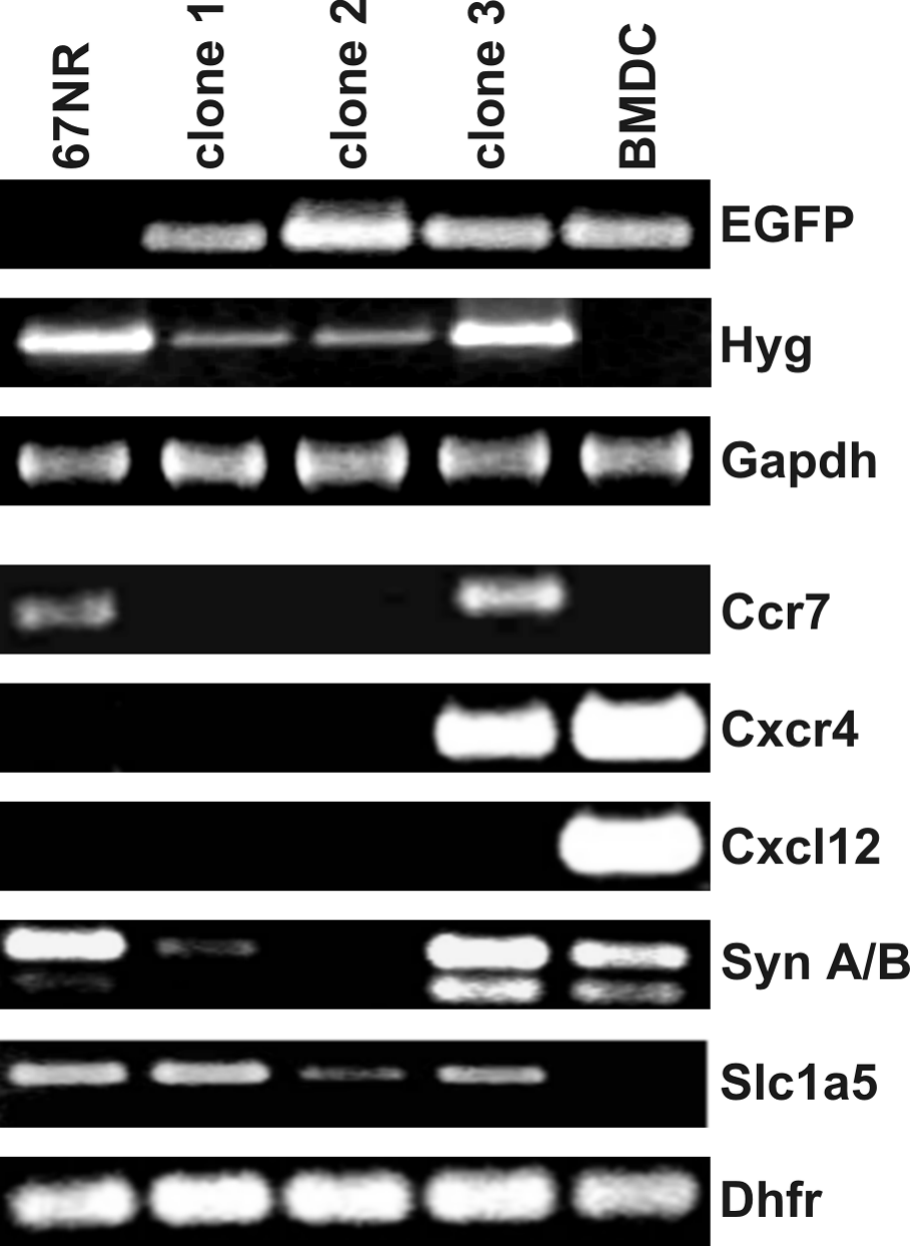
**PCR analysis of parental cells and mBMDC/67NR-Hyg clones**. PCR analysis was performed to investigate whether mBMDC/67NR-Hyg clones originated by cell fusion. EGFP, which was used as a marker for mBMDCs, is expressed in mBMDCs and all mBMDC/67NR-Hyg clones, but not in 67NR-Hyg mouse mammary carcinoma cells. By contrast, the hygromycin resistance gene is detectable in 67NR-Hyg mouse mammary carcinoma cells and mBMDC/67NR-Hyg clones, but not in mBMDCs. In addition to EGFP and hygromycin further molecules have been analyzed by PCR and were found to be differentially regulated among parental cells and mBMDC/67NR-Hyg clones.

In order to prove whether these cell clones originated from putative cell fusion events we first investigated for the expression of parental marker molecules in mBMDC/67NR-clones. Thereby, EGFP was used as mBMDC marker, whereas for 67NR-Hyg cells the hygromycin resistance gene was used. Figure 1 clearly shows that EGFP was expressed in mBMDCs as well as in each mBMDC/67NR-clone, but not in parental 67NR-Hyg mammary carcinoma cells. By contrast, the hygromycin resistance gene was clearly detected in murine 67NR-Hyg mammary carcinoma cells and all mBMDC/67NR-clones, but not in mBMDCs (Figure 1).

In addition to PCR analysis of parental marker molecules we additionally performed short-tandem-repeat (STR) analyses and single nucleotide polymorphism (SNP) analyses. STR analysis was performed for chromosomes 4, 6, 12, 17, and 18, whereas SNP analysis was carried out for chromosomes 1, 3, 5, 11, 13, 16. STR analysis of chromosome 17 was analyzed by conventional PCR since the lengths of the parental alleles were large enough to separate them by conventional DNA gel-electrophoresis. However, only the parental 67NR-Hyg allele was found in all mBMDC/67NR-Hyg clones (Additional File 1). Similar results were obtained for STR analyses of chromosome 12. Both Tg(GFPU)5Nagy/J mice and 67NR-Hyg mouse mammary carcinoma cells possessed different alleles, but mBMDC/67NR-Hyg clones 1-3 carried only the allele of the parental cancer cell line (Additional File 1). By contrast, STR analyses for chromosome 4, 6, and 18 showed that the parental cells possessed identical alleles and thus a discrimination was not feasible.

SNP analysis was informative for marker rs32800995 (chromosome 1), rs3022953 (chromosome 3), rs3023062 (chromosome 5), rs3088673 (chromosome 11), rs3023382 (chromosome 13), and rs3023435 (chromosome 16). Both parental cell lines/strains carried the alleles as given in the mouse genome information SNP database. However, mBMDC/67NR-Hyg clones 1-3 had only alleles in common with the 67NR-Hyg mouse mammary carcinoma cells (Additional File 2).

### mBMDC/67NR-Hyg clones neither exhibited an increased mean chromosomal number nor an increased proliferation rate

Next the mean chromosomal number of mBMDC/67NR-Hyg clones in relation to their parental cells was analyzed. Of interest was the finding that the mean chromosomal number of mBMDC/67NR-Hyg clones was in between the mean chromosomal number of the parental cells, namely 61 ± 19 (clone 1; Table 1), 66 ± 12 (clone 2; Table 1), and 50 ± 8 (clone 3; Table 1).

**Table 1:** Mean chromosomal number of parental cells and its mBMDC/67NR-Hyg clones

Cell line	mean chromosomal number
BMDC	40 ± 0
67NR-Hyg	71 ± 12
clone 1	61 ± 19
clone 2	66 ± 12
clone 3	50 ± 8

**Figure 2:**
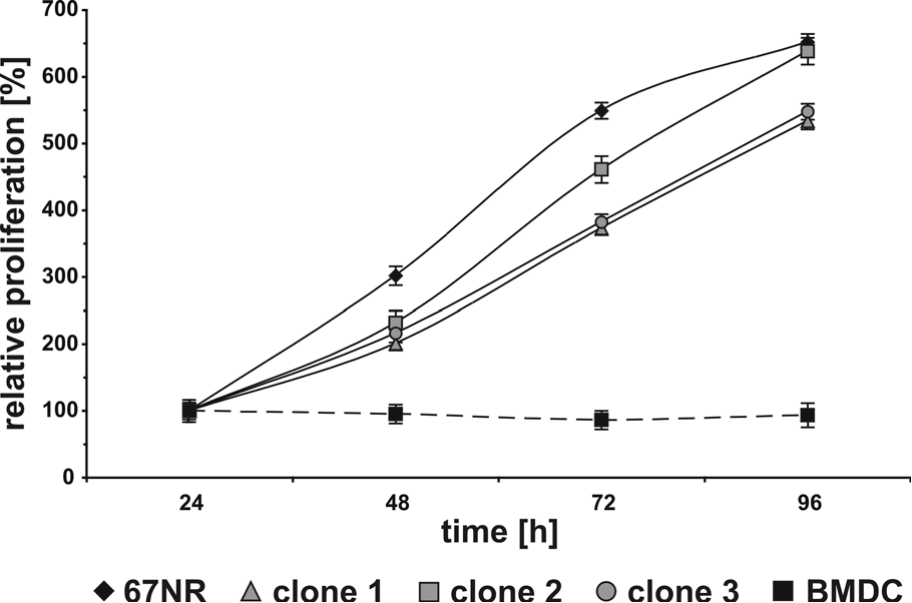
**PCR analysis of parental cells and mBMDC/67NR-Hyg clones**. Proliferation of parental 67NR-Hyg mouse mammary carcinoma cells, mBMDCs, and mBMDC/67NR-Hyg hybrid cell clones 1-3 was assessed by XTT-assay. Shown is the relative proliferation of cells in relation to 24h values, which were set to 100%.

Analysis of the cell proliferation rate indicated that all mBMDC/67NR-Hyg clones exhibited a slightly lower proliferation rate than parental murine 67NR-Hyg cancer cells (Figure 2). Thereby, clone 2 showed the highest proliferatory activity of all clones, whereas the relative cell growth of clone 1 and clone 3 was nearly similar.

### RealTime-PCR array data revealed a dramatic increase in ABC multidrug transporters in mBMDC/67NR-Hyg clones

**Figure 3:**
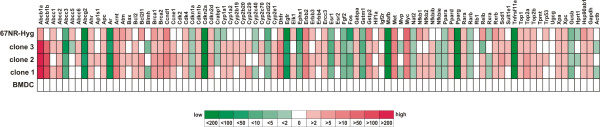
**RealTime PCR analysis of parental cells and mBMDC/67NR-Hyg clones**. Relative gene expression pattern of 67NR-Hyg mouse mammary carcinoma cells, mBMDCs, and mBMDC/67NR-Hyg hybrid cell clones 1-3. Gene expression levels were determined by using the "Mouse Cancer Drug Resistance and Metabolism" RealTime-PCR-array. The expression levels of the appropriate genes of the cancer cell line and mBMDC/67NR-Hyg clones were calculated in relation to the expression level of the appropriate genes of mBMDCs, which were set to 0. Each mBMDC/67NR-Hyg clone exhibits an unique gene expression profile. A difference in the gene expression level of greater than two-fold and less than two-fold was considered as significant.

To investigate whether mBMDC/67NR-Hyg clones exhibited an altered gene expression pattern as compared to their parental cells a RealTime-PCR array analysis was conducted by using the "Mouse Cancer Drug Resistance and Metabolism" array. This array covers 84 targets including genes for drug resistance, drug metabolism, DNA repair, growth factor receptors, and hormone receptors. For a better comparison among the analyzed cell lines the gene expression levels of mBMDC/67NR-Hyg clones and the 67NR-Hyg cell line were calculated in the relation to the gene expression pattern of mBMDCs, which was set to 1. The relative fold-changes of all analyzed genes are shown in Figure 3.

In accordance to previously published data [16] each mBMDC/67NR-Hyg clone possessed a unique gene expression pattern (Figure 3). Thereby, for some genes of a single mBMDC/67NR-Hyg clone the relative expression was in between of those of the parental cells (e.g., Egfr: mBMDC: 1, clone 1: -114-fold, clone 2: -65-fold, clone 3: -59-fold, 67NR-Hyg: -257-fold; Myc: mBMDC: 1, clone 1: +21-fold, clone 2: +12-fold, clone 3: +18-fold, 67NR-Hyg: +50-fold), whereas for other genes the appropriate expression levels were higher/lower in single mBMDC/67NR-Hyg clone as compared to parental cells (e.g., Esr1: mBMDC: 1, clone 1: -5-fold, clone 2: -9-fold, clone 3: -22-fold, 67NR-Hyg: -3-fold).

**Figure 4:**
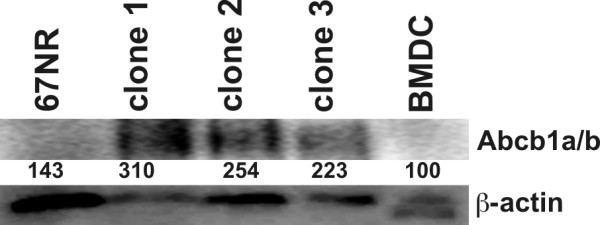
**Western Blot analysis of Abcb1a/b expression**. Western Blot analysis was performed to verify Abcb1a/Abcb1b RealTime-PCR data and clearly show that the ABC multidrug transporters were markedly up-regulated in mBMDC/67NR-Hyg clones. The used antibody recognizes both Abcb1 isoforms. β-actin served as a loading control. Numbers indicate the relative Abcb1a/b expression levels in relation to β-actin, whereby Abcb1a/b expression levels of BMDCs were set to 100%.

Of particular interest was the finding that the expression levels of the ABC multidrug transporters Abcb1a and Abcb1b were markedly increased in all clones as compared to parental cells (Figure 3; Table 2). For instance, the relative expression levels of Abcb1a and Abcb1b were up to +626-fold and up to +113-fold in clone 1 (Figure 3; Table 2). In order to verify RealTime-PCR array data for Abcb1a/Abcb1b Western Blot analysis was performed. Data are summarized in Figure 4 and clearly depict the marked upregulation of Abcb1a/Abcb1b in mBMDC/67NR-Hyg clones 1 to 3. Determination of the relative intensities of Abcb1a/b expression levels in comparison to β-actin expression levels in parental cells and hybrid cells nicely fitted to the RealTime-PCR array data (Figure 4).

**Table 2:** ABC multidrug transporter expression

	BMDC	clone 1	clone 2	clone 3	67NR-Hyg
Abcb1a	1	**626**	**373**	**566**	**8**
Abcb1b	1	**113**	**97**	**40**	1
Abcc1	1	**2**	1	**2**	**2**
Abcc2	1	**5**	**3**	**4**	1
Abcc3	1	*-28*	*-24*	*-94*	*-18*
Abcc5	1	-1	-1	*-2*	**2**
Abcc6	1	1	**3**	**3**	1
Abcg2	1	*-117*	*-79*	*-92*	*-200*
Mvp	1	*-2*	1	*-3*	1

In addition to EGFP (marker for mBMDCs) and hygromycin resistance (marker for 67NR-Hyg), we further analyzed the expression of Ccr7, Cxcr4, Cxcl12, syncytin A (Syn A) and syncytin B (Syn B), and Slc1A5 in both parental cells as well as in all mBMDC/67NR-Hyg clones. Ccr7 (binds Ccl19 and Ccl21) and Cxcr4 (binds Cxcl12 (also named stromal cell-derived factor 1α)) have been associated with the organ specific metastatic spreading of breast cancer cells (for review see [39]), while syncytin A, syncytin B and its receptor Slc1A5 [25] have been linked to cell fusion

Ccr7 was expressed in parental 67NR-Hyg cells and hybrid clone 3, but not in mBMDCs and clone 1 and 2 (Figure 1). By contrast, Cxcr4 was solely expressed in mBMDCs and clone 3, but neither in 67NR-Hyg carcinoma cells nor in clone 1 and 2 (Figure 1). Whether this indicates that clone 3 may exhibit an enhanced capability to preferentially metastasize into regional lymph nodes as well as, e.g., bone marrow or liver, remains to be elucidated. Cxcl12 was solely expressed in mBMDCs (Figure 1). A differential expression was also observed for Syn A and Syn B as well as for their receptor Slc1A5. Syn A and Syn B were markedly expressed in mBMDCs and clone 3, whereas 67NR-Hyg predominantly expressed Syn A and only lower amounts of Syn B (Figure 1). Clone 2 was negative for both Syn A and Syn B, whereas clone 1 was weakly positive for Syn A (Figure 1). Slc1a5 was expressed by 67NR-Hyg cells and all three mBMDC/67NR-Hyg clones, whereas mBMDCs were negative for this receptor (Figure 1).

### Increased ABC multidrug transporter levels correlate with a marked Rhodamine 123 efflux

**Figure 5:**
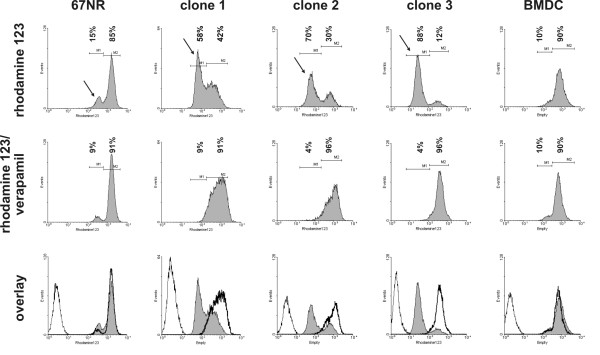
**Rhodamine 123 is efficiently effluxed in mBMDC/67NR-Hyg clones**. Rhodamine 123 efflux was assayed to prove whether Abcb1a/Abcb1b up-regulation in mBMDC/67NR-Hyg clones was functional. Cells were loaded with Rhodamine 123 and analyzed for the remaining cellular Rhodamine 123 content after 40 minutes by flow cytometry. The side-population (SP) population, representing cells, which have effectively effluxed Rhodamine 123, are marked by an arrow. Verapamil was used to specifically block Abcb1a/Abcb1b multidrug transporters. Flow cytometry data revealed that Rhodamine 123 is efficiently effluxed in mBMDC/67NR-Hyg clones; an effect, which was completely blocked by verapamil.

In order to investigate whether the altered expression levels of Abcb1a/Abcb1b and Abcg2 were associated with a different phenotype of the cells, the efflux of Rhodamine 123 was investigated. In accordance with RealTime-PCR array data and Western Blot analysis mBMDCs were not capable to efflux Rhodamine 123 indicated by a bright Rhodamine 123 staining (Figure 5). By contrast, a slight population of weakly Rhodamine 123 stained 67NR-Hyg cells, the so-called side-population (SP) fraction, was clearly detectable (Figure 5), which is in accordance to RealTime-PCR array data showing that Abcb1a was nearly 8-fold higher expressed in 67NR-Hyg cells as compared to mBMDCs. Moreover, inhibition of ABC multidrug transporter activity by verapamil resulted in a markedly decreased 67NR-Hyg SP fraction (Figure 5).

In contrast to parental cells the SP fraction was the main population among mBMDC/67NR-Hyg clones, whereby Rhodamine 123 was most efficiently effluxed by clone 3. Here, nearly 88% of the cells showed low-intensity fluorescence of Rhodamine 123 (Figure 5). Inhibition of Abcb1a/b activity by verapamil completely blocked the Rhodamine 123 efflux in all clones (Figure 5).

### Increased ABC multidrug transporter levels correlate with a resistance of clones towards chemotherapeutic compounds

To investigate whether the markedly increased Abcb1a/b expression levels were correlated to an increased resistance of mBMDC/67NR-Hyg clones towards cytotoxic compounds XTT cytotoxicity assays were carried out. Thereby, mBMDC/67NR-Hyg clones and parental cells were cultured for up-to three days in the presence of different concentrations of 5-Fluorouracil (5-FU), 17-DMAG, doxorubicin, etoposide, and paclitaxel. Likewise, cells were cultured in the presence of 1 μM of the appropriate chemotherapeutic compound and 50 μM verapamil to explore whether resistance was mediated by Abcb1a/b multidrug transporters.

**Figure 6:**
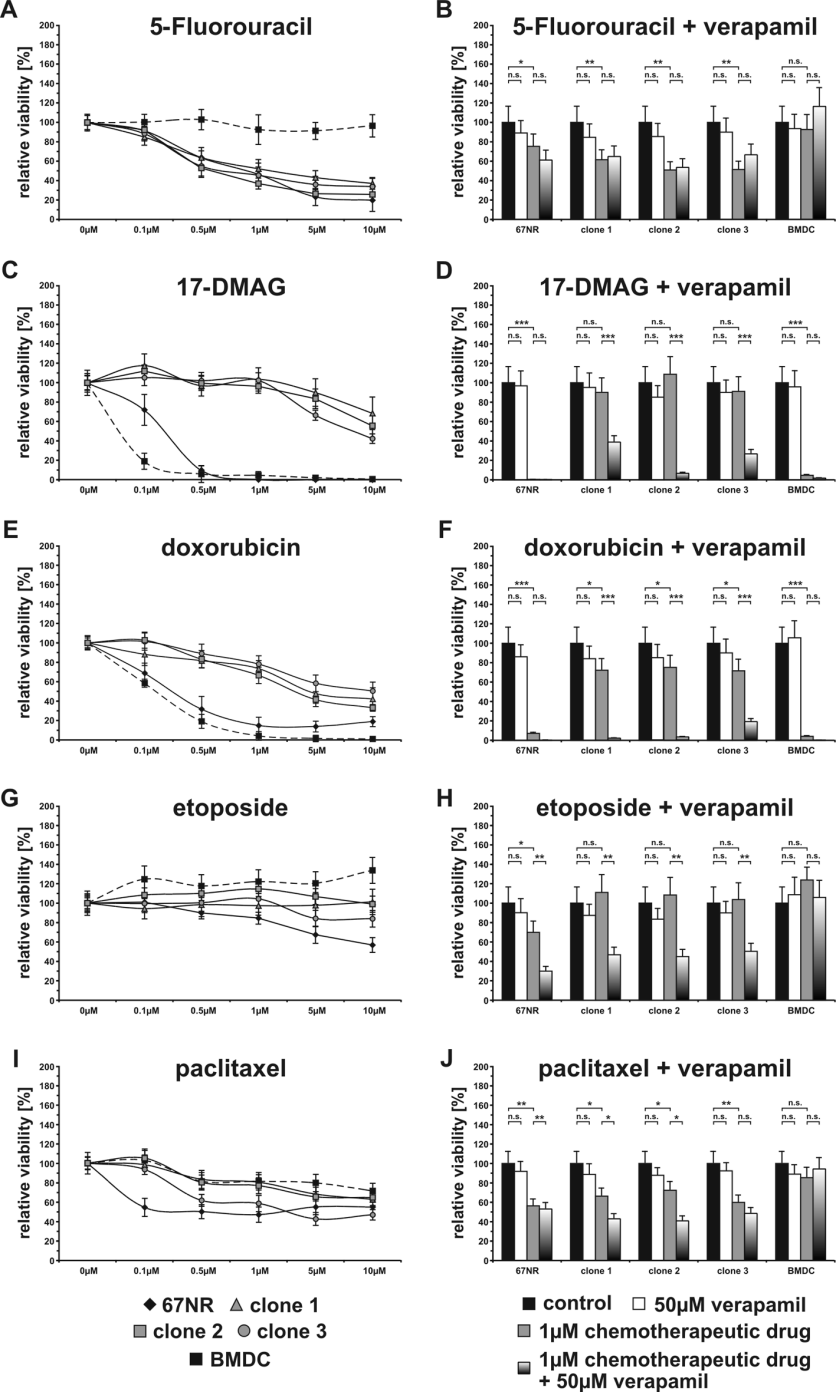
**mBMDC/67NR-Hyg clones exhibit an enhanced resistance towards several chemotherapeutic drugs**. XTT proliferation assay was conducted to investigate whether the increased Abcb1a/Abcb1b expression levels of the mBMDC/67NR-Hyg clones were correlated with an enhanced drug resistance. mBMDC/67NR-Hyg clones showed a marked resistance towards 17-DMAG and doxorubicin as well as etoposide and paclitaxel, but not 5-Fluorouracil. Inhibition of Abcb1a/Abcb1b activity by verapamil completely blocked the doxorubicin resistance of mBMDC/67NR-Hyg clones. By contrast, resistance of mBMDC/67NR-Hyg clones towards 17-DMAG, etoposide, and paclitaxel was partially impaired by verapamil indicating that additional ABC transporters or other drug resistance mechanisms are involved in the mBMDC/67NR-Hyg clones resistance towards these chemotherapeutic compounds.

In accordance to previously published data [16] mBMDC/67NR-Hyg clones did not exhibit an overall resistance, but rather an enhanced resistance towards certain chemotherapeutic drugs, including 17-DMAG, doxorubicin, etoposide, and paclitaxel (Figure 6C, 6E, 6G, 6I). In contrast to this, mBMDC/67NR-Hyg clones did not show an enhanced resistance towards 5-FU (Figure 6A). Here, the survival rate of cells was comparable to the survival rate of parental 67NR-Hyg cells (Figure 6A). Interestingly, mBMDCs grew well in the presence of 5-FU without any dose-dependent cytotoxic effect (Figure 6A). Inhibition of Abcb1a/b multidrug transporter activity by verapamil had no effect on the proliferation rate of hybrid cells and parental cells (Figure 6B) suggesting that resistance of mBMDCs towards 5-FU is not mediated by Abcb1a/b multidrug transporters.

By contrast, both mBMDCs and 67NR-Hyg mouse mammary cancer cells showed a high susceptibility towards 17-DMAG and doxorubicin (Figure 6C, 6E). In case of 17-DMAG concentrations of 0.1 μM (mBMDCs) and 0.5 μM (67NR-Hyg) were sufficient to effectively kill all parental cells (Figure 6C). By contrast, the proliferation of parental cells was impaired by doxorubicin in a dose-dependent manner (Figure 6E). All mBMDC/67NR-Hyg clones possessed a marked resistance towards these two chemotherapeutic compounds (Figure 6C, 6E). Concentrations of up to 5 to 10 μM 17-DMAG were recommended to ultimately kill mBMDC/67NR-Hyg clones (Figure 6C), whereas the proliferation of mBMDC/67NR-Hyg cells was impaired by doxorubicin in a dose-dependent manner (Figure 6E). Resistance of cells towards both 1 μM 17-DMAG and doxorubicin was significantly blocked by 50 μM verapamil suggesting that the resistance of mBMDC/67NR-Hyg clones towards these cytotoxic compounds is facilitated by Abcb1a/b multidrug transporters (Figure 6D, 6F).

In accordance to 17-DMAG and doxorubicin, mBMDC/67NR-Hyg clones also exhibited a marked resistance towards etoposide (Figure 6G). Of interest was the effect of etoposide on mBMDCs, whose proliferation rate was slightly increased by this drug, whereby a dose-dependent effect was not observed (Figure 6G). Inhibition of Abcb1a/b multidrug transporter activity by verapamil had only a partial effect (Figure 6H). Compared to mBMDC/67NR-Hyg cells treated with 1 μM etoposide growth of etoposide and verapamil co-treated cells was solely, but significantly, decreased by about 50 to 60% (Figure 6H). This suggests that resistance of mBMDC/67NR-Hyg clones towards etoposide was solely partially facilitated by Abcb1a/b multidrug transporters.

We also observed a slight resistance of mBMDC/67NR-Hyg cells towards paclitaxel, whereby the proliferation rate of clone 1 and clone 2 within the presence of different paclitaxel concentrations was comparable to parental mBMDCs (Figure 6I). In contrast to this, clone 3 revealed a weaker resistance towards paclitaxel (Figure 6I). The differential resistance of mBMDC/67NR-Hyg clones towards paclitaxel was also apparent when inhibiting Abcb1a/b multidrug transporter activity by verapamil. In case of clone 1 and clone 2 resistance towards paclitaxel was slightly significantly impaired by 50 μM verapamil, whereas verapamil had no effect on the cell growth of clone 3 within the presence of 1 μM paclitaxel (Figure 6J).

## Discussion

In the present study we investigated the possible outcome of co-cultivation of murine mammary 67NR-Hyg breast cancer cells and murine BMDCs derived from Tg(GFPU)5Nagy/J mice [35]. The rationale of this study was given by the fact that tumor tissue resembles chronically inflamed tissue (tumors are often described as wounds that do not heal) [1,2,40] and that chronic inflammation is a strong stimulus for the recruitment of BMDCs [3-5]. This correlation suggests that the recruitment of BMDCs into tumor tissues is a common process.

Here, co-cultivation of murine BMDCs with 67NR-Hyg mouse mammary carcinoma cells resulted in the origin of cells exhibiting markedly increased expression levels of the ABC multidrug resistance transporters Abcb1a and Abcb1b concomitant with an enhanced resistance towards chemotherapeutic drugs.

The finding that each of the analyzed mBMDC/67NR-Hyg clones was positive for EGFP suggests that the clones have originated by cell fusion. By contrast, both STR and SNP analyses revealed that only parental 67NR-Hyg alleles were present in mBMDC/67NR-Hyg clones, thus facing the question whether mBMDC/67NR-Hyg clones rather originated by horizontal gene transfer (HGT). 67NR-Hyg cells might have uptaken DNA containing microvesicles or apoptotic bodies shedded from mBMDCs. In a recent work Ehnfors and colleagues demonstrated in an *in vivo *setting that endothelial cells can take up tumor cell DNA via HGT [33] suggesting that a transfer of mBMDC DNA to 67NR-Hyg cells by HGT should be feasible. On the other hand, HGT is a random process. It can not be predicted which DNA fragments are embedded in microvesicles/apoptotic bodies, which of these vesicles/bodies will be uptaken by target cells and whether the uptaken DNA will be ultimately incorporated into the genome of the target cells. Because of that it remains ambiguous why all mBMDC/67NR-Hyg clones were positive for EGFP.

Another argument which would favor HGT as the mechanism of mBMDC/67NR-Hyg clone origin might be the fact that the mean chromosomal number of the clones was not the sum of the parental chromosomes. The mean chromosomal number of mBMDC/67NR-Hyg clones varied between 50 ± 8 to 66 ± 12 chromosomes, which was rather half of the sum of the mean chromosomal number of the parental cells. In fact, recent studies demonstrated that cell fusion commonly went along with a summation of parental chromosomes in hybrid cells [16,17,41]. For instance, we have recently demonstrated that breast epithelial/breast cancer hybrid cells, which derived from spontaneous fusion events, possessed a mean chromosomal number of 78 ± 11 (M13MDA435 clone 1) to 88 ± 13 (M13MDA435 clone 2), which was nearly the sum of the parental mean chromosomal number [20]. On the contrary, the transition of a hybrid cell from a heterokaryon (two nuclei) to a synkaryon (one nucleus) is generally associated with a loss of chromosomes [19]. Likewise, the aneuploid karyotype of most hybrid cells is unstable and because of that chromosomes are unequally segregated or even lost during further cell divisions - a process, which has been termed autocatalytic karyotypic evolution [42]. Another possibility that could explain the phenomenon of a reduced mean chromosomal number in mBMDC/67NR-Hyg clone might be ploidy reduction [43]. This process has been recently reported for murine fusion-derived hepatocytes [43], whereby the mechanisms that direct such a "meiosis-like" effect in somatic cells still remains elusive. In any case, the authors observed a high degree of marker loss in diploid daughter cells indicating that ploidy reductions lead to the generation of highly genetically diverse daughter cells with about 50% reduction in nuclear content [43,44]. This dynamic model of hepatocyte polyploidization, ploidy reversal and aneuploidy (which has been referred to as "ploidy conveyor") has likely evolved to generate genetic diversity, thereby permitting adaptation of hepatocytes to xenobiotic or nutritional injury [44]. In context of our study, such a mechanism would be a suitable explanation for the finding that mBMDC/67NR-Hyg clones possessed a reduced mean chromosomal number concomitant with an increased drug resistance.

Thus, the question whether mBMDC/67NR-Hyg clones ultimately originated by cell fusion or by HGT can not be answered sufficiently. Both mechanisms are conceivable. Recent data of Duncan et al. let assume that the analyzed mBMDC/67NR-Hyg cells might have originated by cell fusion accompanied by subsequent ploidy reduction and autocatalytic karyotypic evolution. Nonetheless, HGT can not be ruled out completely.

The cytotoxicity data presented here nicely fitted to the ABC multidrug transporter expression levels of the analyzed cells. Drug efflux is chiefly mediated by ABC multidrug transporter(s), whereby a single drug can be exported by several ABC multidrug transporters, and each ABC multidrug transporter can confer characteristics resistance pattern to cells [45]. By using the power of combination knockout mice, being deficient for distinct murine ABC multidrug transporters, Lagas et al. provided an update for murine ABC multidrug transporter and substrate specificities [46,47]. Thereby, etoposide resistance is mediated by Abcb1a/b, Abcc1, Abcc2, and Abcc3, whereas resistance to both doxorubicin and paclitaxel is facilitated by Abcb1a/b and Abcc2 [46,47]. Thus the increased Abcb1a/b expression levels of mBMDC/67NR-Hyg clones correlate well to the cells resistance towards doxorubicin, which is further strengthen by reverting the cells resistance with the ABC1B (PGP/MDR1) inhibitor verapamil. Data showing that resistance towards 17-DMAG (clone 1 and clone 3), etoposide and paclitaxel was solely partially blocked by verapamil suggests that further ABC multidrug transporter(s) or other mechanism do contribute to the cells resistance towards these chemotherapeutic compounds. Paclitaxel resistance has been associated with Abcb1a/b and Abcc2 [47]. Since all mBMDC/67NR-hyg clones possessed slightly higher Abcc2 expression levels and Abcc2 has been reported to be the main transporter for biliary excretion [47] it can be assumed that Abcc2 contribute to paclitaxel resistance in the presence of verapamil. By contrast, Abcb1a/b are the main transporters for biliary excretion of doxorubicin, and Abcc2 only partly compensates for the absence of Abcb1a/b [47], which nicely correlates with the finding that doxorubicin resistance of mBMDC/67NR-Hyg clones was completely abrogated by verapamil. Likewise, etoposide resistance has been correlated to Abcb1a/b, Abcc1, Abcc2, and Abcc3 [46,47]. Since only Abcc2 was slightly higher expressed in mBMDC/67NR-Hyg clones as compared to parental murine 67NR-Hyg carcinoma cells, which further correlates with the relative survival rates of mBMDC/67NR-Hyg clones in comparison to 67NR-Hyg cells, we conclude that etoposide is also effluxed by this ABC multidrug transporter. Transfection of mouse fibroblasts with murine Abcg2 resulted in an increased etoposide resistance of the cells [48] suggesting that also the increased Abcg2 levels in mBMDC/67NR-Hyg clones might have contributed to the enhanced etoposide resistance.

In addition to the basal ABC multidrug resistance transporter expression levels further mechanisms might also contribute to the enhanced drug resistance of mBMDC/67NR-Hyg clones. RealTime-PCR-array data showed that several cytochrome p450 family members were slightly higher expressed in mBMDC/67NR-Hyg clones than in parental cells suggesting that drug inactivation might be another mechanism. Likewise, due to the aneuploid karyotype mBMDC/67NR-Hyg cells will be able to adapt to the cytotoxic conditions. This assumption is in view with preliminary data showing that mBMDC/67NR-Hyg clones growing in 10 μM doxorubicin exhibited an altered phenotype, such as an increased cell diameter.

## Conclusions

Our data indicate that co-cultivation of mBMDCs and murine 67NR-Hyg mammary carcinoma cells can give rise to highly drug resistant mBMDC/67NR-Hyg cells. Even though it remains unknown whether these cells originated by HGT or cell fusion our data might reflect to the fatal impact of inflammation in the context of cancer. Tumor tissue resembles chronically inflamed tissues resulting in the recruitment of BMDCs. Moreover, conventional cancer therapies, such as chemotherapy and radiation, do also induce a strong inflammatory response within the tumor tissues concomitant with BMDC recruitment due to massive tumor cell destruction. Irrespective of how genetic information is exchanged between two cells/two cell types: this process could end in the evolution of a new cell/cell type exhibiting novel properties, such as an enhanced metastatogenic capacity or an enhanced drug resistance.

## Materials and methods

### Cell culture and transfection

The murine mammary carcinoma cell line 67NR was purchased from the American Tissue Culture Collection (ATCC, LGC Standards GmbH, Wesel, Germany) and was maintained in Dulbecco's Modified Eagle's Medium (DMEM, PAA, Linz, Austria) supplemented with 10% fetal calf serum (FCS; PAA, Linz, Austria) and 1% Penicillin/Streptomycin (100 U/ml Penicillin, 0.1 mg/ml Streptomycin; PAA Laboratories, Linz, Austria) at 37°C and 5% CO_2 _in a humidified atmosphere. Stable transfection of 67NR cells with the pKS-Hyg plasmid was done by electroporation using the AMAXA Nucleofector Technology (Lonza Cologne AG, Cologne, Germany) in accordance to the manufacturers' instructions. Hygromycin resistant cells were selected by addition of 200 μg/ml Hygromycin B (PAA Laboratories, Linz, Austria) to the media.

Murine bone marrow-derived cells (mBMDCs) were obtained from the femurs of 8 - 9 week-old female transgenic Tg(GFPU)5Nagy/J mice (Jackson Laboratories, Bar Habor, Maine, USA) expressing the enhanced green fluorescent protein (EGFP) and the puromycin-resistance gene [35]. Bone marrow cells were collected by flushing the bone shaft with Iscove's Modified Dulbecco's Medium (IMDM; PAA, Linz, Austria) using a syringe and a 26G needle. The cell suspension was seeded into a 75 cm^2 ^tissue culture flask and cultivated for 24h in DMEM (PAA, Linz, Austria) supplemented with 10% FCS (PAA, Linz, Austria) and 1% Penicillin/Streptomycin (100 U/ml Penicillin, 0.1 mg/ml Streptomycin; PAA Laboratories, Linz, Austria) at 37°C and 5% CO_2 _in a humidified atmosphere. Subsequently, non-adherent cells were removed and the remained mBMDCs were cultivated for additional two weeks. Cells were passaged once prior for their use in experiments.

### Co-cultivation of mBMDC and 67NR-Hyg mammary carcinoma cell

Murine BMDCs (1 × 10^6^) and 67NR-Hyg mouse mammary cancer cells (1 × 10^6^) were co-cultivated for 24h in DMEM (PAA, Linz, Austria) supplemented with 10% FCS (PAA, Linz, Austria) and 1% Penicillin/Streptomycin (100 U/ml Penicillin, 0.1 mg/ml Streptomycin; PAA Laboratories, Linz, Austria) without Hygromycin and Puromycin at 37°C and 5% CO_2 _in a humidified atmosphere. After 24h both antibiotics were added to the media (Hygromycin B: 200 μg/ml; Puromycin: 5 μg/ml; Puromycin was purchased from Sigma Aldrich, Taufkirchen, Germany). Double resistant clones were first analyzed for a green fluorescence, then isolated and cultivated separately from each other. Isolated clones were named as mBMDC/67NR-clone-X, whereby "X" marks the clone number.

### RT-PCR

RNA was isolated from 1 × 10^6 ^cells by using the NucleoSpin^® ^RNA II Kit (Macherey-Nagel GmbH, Düren, Germany) in accordance to the manufacturers' instructions. Reverse Transcription of RNA into cDNA was performed using the RevertAid^TM ^First Strand cDNA Synthesis Kit (Fermentas, St. Leon-Rot, Germany) as referred to the instruction manual. PCR was performed in a 25 μl reaction mixture containing 1.25U Taq Polymerase, 1 × reaction buffer, 2 mM MgCl2, 200 μM of each dNTP (all reagents were purchased from Fermentas, St. Leon-Rot, Germany) and 100 pM primers (Invitrogen, Karlsruhe, Germany). The cycling conditions comprised of an initial denaturation of 5 min at 94°C and 30 cycles of 0.5 min at 94°C, 0.5 min at the appropriate annealing temperature and 0.5 min at 72°C followed by a final elongation for 10 min at 72°C. The used primer pairs concomitant with their specific annealing temperature and product lengths are summarized in Table 3.

**Table 3:** Summary of primer pairs for PCR

Name	Annealing temperature	Mean product size	Primer	Sequence (5' to 3')
Ccr7	61°C	208 bp	forward	AGCACCATGGACCCAGGGA
			reverse	CTGCCTCTCATGTATTCTGT
Cxcr4	57°C	390 bp	forward	GGCTGTAGAGCGAGTATTGC
			reverse	GTAGAGGTTGGTGACAGTGTAGAT
Cxcl12	61°C	512 bp	forward	ACACTCCGCCATAGCATATGGT
			reverse	TGAAGCATGCGTTTGGAGG
EGFP	61.5°C	220 bp	forward	GACAAGCAGAAGAACGGCATCAAG
			reverse	CGGCGGCGGTCACGAACT
HYG	60.5°C	500 bp	forward	AGCTGCGCCGATGGTTTCTACAA
			reverse	ATCGCCTCGCTCCAGTCAATG
Syn A	60°C	281 bp	forward	TACCTGATGCGCCTGGAGCT
			reverse	AAGCTTTGCAGGAACTGGAGAA
Syn B	60°C	201 bp	forward	CCACCACCCATACGTTCAAA
			reverse	GGTTATAGCAGGTGCCGAAG
Slc1a5	59°C	585 bp	forward	CTGGATTATGTGGTACGCCAC
			reverse	GACCTGTCCACTAGCCAGTC
Dhfr	59°C	159 bp	forward	CCACAACCTCTTCAGTGGAAGGTAAACAGA
			reverse	TTGGCAAGAAAATGAGCTCCTCGTGG
Gapdh	68°C	980 bp	forward	TGAAGGTCGGTGTGAACGGATTTGGC
			reverse	CATGTAGGCCATGAGGTCCACCAC

### Short tandem repeat (STR) and Single nucleotide polymorphism (SNP) analysis

Genomic DNA was extracted by using the NucleoSpin^® ^Tissue Kit (Macherey-Nagel GmbH, Düren, Germany) in accordance to the manufacturers' instructions. Amplification of DNA fragments for STR and SNP analysis was performed by conventional PCR according to above-mentioned protocol, whereby 25 ng genomic DNA were used as a template. Suitable STRs and SNPs for analysis were determined in accordance to the mouse genome information strains, SNPs & polymorphisms database (http://www.informatics.jax.org/strains_SNPs.shtml) and the genetic quality control annual report (http://jaxmice.jax.org/geneticquality/gqcreport.pdf). The used primer pairs and product lengths are summarized in additional file 3 and additional file 4. For STR analysis PCR products (1 μl) were mixed with 9.5 μl Hi-Di^TM ^Formamide and 0.5 μl GeneScan™-500 LIZ^® ^size standard (Applied Biosystems, Darmstadt, Germany) in a 96-well microtiter plate. After heating the loading cocktail for 3 min at 95°C samples were immediately chilled on ice. Samples were analyzed by capillary electrophoresis for 2,500s by using a 3130 × l ABI PRISM Genetic Analyzer (Applied Biosystems, Darmstadt, Germany). Data analysis was performed with the GeneMapper (v 4.0) software (Applied Biosystems, Darmstadt, Germany). For SNP analysis PCR products were purified with the QIAquick^®^PCR purification kit (QIAGEN, Hilden, Germany) as recommended. PCR products (1-10 ng) were sequenced with the BigDye^®^Terminator v1.1 Cycle Sequencing Kit (Applied Biosystems, Darmstadt, Germany) in accordance to the manufacturer's instructions. Not incorporated labeled ddNTPs were removed with the DyeEx^® ^2.0 spin kit (QIAGEN, Hilden, Germany) as described in the manual. Purified sequencing products were analyzed by using a 3130 × l ABI PRISM Genetic Analyzer (Applied Biosystems, Darmstadt, Germany). Data analysis was performed with the ABI PRISM 3130 data collection software (Applied Biosystems, Darmstadt, Germany) and the ClustalW2-Multiple Sequencing Alignment tool (European Bioinformatics Institute, Wellcome Trust Genome Campus, Hinxton, UK).

### Chromosome spreading

Cells (1 × 10^6^) were cultivated with 0.2 μg/ml colcemid (Sigma Aldrich, Taufkirchen, Germany) for 4 - 6h. Subsequently, cells were harvested, washed once with PBS, and were carefully resuspended in 10 ml 75 mM KCl. After 30 min the cells were sedimented (160 × g, 10 min) and the supernatant was discarded. Cells were carefully resuspended in the remaining KCl solution. Hereafter, 10 ml methanol/acetic acid solution (3:1; both chemicals were purchased from Sigma Aldrich, Taufkirchen, Germany) was added dropwise under continuous stirring to the cells. Cells were then washed at least twice in methanol/acetic acid solution. Finally, the methanol/acetic acid fixed cells were dropped onto a H_2_O wetted cover slip. To visualize spread chromosomal DNA cover slips were air-dried and subsequently stained with Sytox Green (Invitrogen, Karlsruhe, Germany) as recommended to the manufacturers' instructions combined with confocal laser scanning microscopy (Leica TCS SP5; Leica, Bensheim, Germany).

### RealTime-PCR

RNA was isolated from 2-4 × 10^6 ^cells by using the NucleoSpin^® ^RNA II Kit from Macherey-Nagel (Macherey-Nagel GmbH, Düren, Germany) in accordance to the manufacturers' instructions. Reverse Transcription of RNA into cDNA was performed using the RT^2 ^First Strand Kit (QIAGEN GmbH, Hilden, Germany) as referred to the instruction manual. In this study, the RT^2^*Profiler*^TM ^PCR Array "Mouse Cancer Drug Resistance and Metabolism" (QIAGEN GmbH, Hilden, Germany) covering 84 genes was applied by using an Applied Biosystems 7700 RealTime-PCR cycler (Applied Biosystems, Darmstadt, Germany). RealTime-PCR was performed using the RT^2 ^SYBR Green Master Mix (QIAGEN GmbH, Hilden, Germany) according to the manufacturers' protocol under the following cycler conditions: 95°C: 10 min; 40 Cycles (95°C: 15s; 60°C: 60s). RealTime-PCR data were analyzed by the 2^-ΔCT ^method on a Microsoft^TM ^Excel^® ^template provided by the manufacturer (QIAGEN GmbH, Hilden, Germany). Significant changes in gene expression among the analyzed cell lines were defined as at least 2-fold up- or downregulation of genes.

### Western Blot

#### Western Blot analysis

To verify RealTime-PCR data for Abcb1a and Abcb1b expression cells (5 × 10^5^) were lysed in SDS sample buffer for 1h at 36°C. Subsequently, samples were separated by SDS-PAGE on a 8% SDS polyacrylamide gel and transferred to PVDF membranes (Millipore, Schwalbach, Germany) under semi-dry conditions. Membranes were blocked overnight with 10% (w/v) non-fat dry milk in TBS-T. Abcb1a/Abcb1b and β-actin were detected by using the following primary antibodies: MDR (Abcb1a/Abcb1b; clone C-19; Santa Cruz Biotechnology, Heidelberg, Germany) and β-actin (clone 13E5; rabbit monoclonal, Cell Signaling, New England Biolabs, Frankfurt am Main, Germany). For detection of primary antibodies the HRP-conjugated secondary anti-rabbit IgG (Cell Signaling, New England Biolabs, Frankfurt am Main, Germany) was used. Bands were visualized using the LumiGLO^® ^Reagent (Cell Signaling, New England Biolabs, Frankfurt am Main, Germany) in accordance to the manufacturers' instructions and were detected with the Aequoria Macroscopic Imaging system (Hamamatsu Photonics Germany, Herrsching am Ammersee, Germany). Relative densities of Abcb1a/b expression in relation to β-actin was determined by using the ImageJ software (http://rsb.info.nih.gov/ij/).

#### Flow cytometry

The efflux of Rhodamine 123 (Sigma Aldrich Taufkirchen, Germany) was analyzed by using a FACScalibur flow cytometer (Becton Dickenson, Heidelberg, Germany). Cells (5 × 10^5^) were incubated in PBS containing 200 μg/ml Rhodamine 123, 50 μM verapamil (Sigma Aldrich Taufkirchen, Germany), or a combination of both for 20 min at 37°C. Thereafter, cells were washed once with PBS and incubated for additional 20 min at 37°C. Rhodamine 123 fluorescence was detected using the FL1-H channel.

#### XTT cytotoxicity assay

The XTT cytotoxicity assay was performed as described [49] with slight modifications. In brief, cells (1 × 10^3^/well) were seeded in triplicates in a 96-well flat-bottom microtiter plate in 0.25 ml of the appropriate culture medium. After 24 h media was replaced by culture media containing different concentrations of either 5-Fluorouracil (5-FU), 17-DMAG, doxorubicin, etoposide or paclitaxel (all drugs were purchased from Sigma Aldrich, Taufkirchen, Germany). In case of verapamil, 1 μM of the appropriate chemotherapeutic drug was used alone or in combination with 50 μM verapamil (Sigma Aldrich, Taufkirchen, Germany). After 48h media was removed and the plates were analyzed with XTT reagent (Roche Diagnostics, Mannheim, Germany) according to the manufacturer's instructions. The absorption of the formed XTT-formazan derivative was measured using a BioTek EL800 microplate reader (BioTek, Bad Friedrichshall, Germany). The EGFP fluorescence of mBMDCs and mBMDC/67NR-Hyg hybrids did not interfere with the XTT-formazan formed derivative. Statistical significance was calculated using Student's *t*-test: n.s. = not significant; * = *P  *< 0.05; ** = *P  *< 0.01; *** = *P  *< 0.001;

## Competing interests

The authors declare that they have no competing interests.

## Authors' contributions

CN performed the experiments. CH performed the STR and SNP analyses and corrected the manuscript. KSZ wrote and corrected the manuscript. TD designed the experiments, wrote and corrected the manuscript. All authors have read and approved the final manuscript.

## Supplementary Material

Additional file 1 **Figure S1: STR analysis of mBMDC/67NR-Hyg clones. A) **STR analysis of chromosome 17 by conventional PCR. Only the parental 67NR-Hyg allele was found in mBMDC/67NR-Hyg clones. Fragment lengths are indicated. **B) **Result of STR analysis of chromosomes 4, 6, 12, and 18 by capillary electrophoresis. Parental cells were homozygote for chromosome 4, 6 and 18 and heterozygote for chromosome 12. However, only the parental 67NR-Hyg chr. 12 allele was present in mBMDC/67NR-Hyg clones. Click here for file

Additional file 2 **SNP analysis of mBMDC/67NR-Hyg clones**. Primer sequences appear in blue. Known polymorphisms are shown in red, whereas unknown polymorphisms are marked in green. SNPs located on chromosome 1, 3, 5, 11, 13 and 16 were analyzed. However, only the parental 67NR-Hyg SNPs were present in mBMDC/67NR-Hyg clones. Click here for file

Additional file 3 Summary of primer pairs for STR analysis Click here for file

Additional file 4 Summary of primer pairs for SNP analysis Click here for file
