# Supplementary material for: Co-cultivation of murine BMDCs with 67NR mouse mammary carcinoma cells give rise to highly drug resistant cells
Source: Cancer Cell Int. 2011 Jun 28;11:21. doi: 10.1186/1475-2867-11-21 (PMC3135493; doi:10.1186/1475-2867-11-21)
Supplement: Additional file 1 — Figure S1: STR analysis of mBMDC/67NR-Hyg clones. A) STR analysis of chromosome 17 by conventional PCR. Only the parental 67NR-Hyg allele was found in mBMDC/67NR-Hyg clones. Fragment lengths are indicated. B) Result of STR analysis of chromosomes 4, 6, 12, and 18 by capillary electrophoresis. Parental cells were homozygote for chromosome 4, 6 and 18 and heterozygote for chromosome 12. However, only the parental 67NR-Hyg chr. 12 allele was present in mBMDC/67NR-Hyg clones. [file 1475-2867-11-21-S1.DOC]

**Additional File 1**


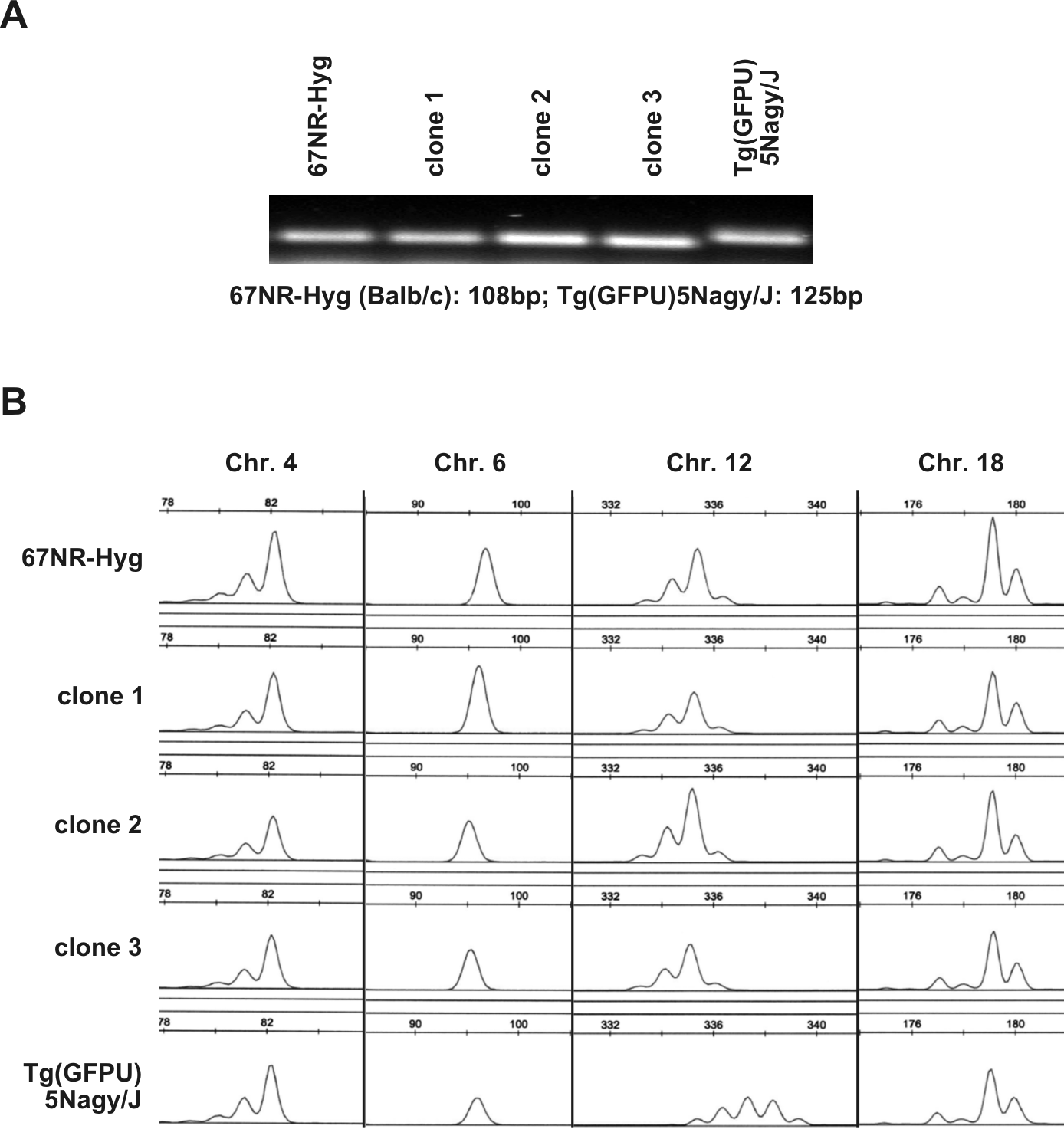


**Figure S1: STR analysis of mBMDC/67NR-Hyg clones. A)** STR analysis of chromosome 17 by conventional PCR. Only the parental 67NR-Hyg allele was found in mBMDC/67NR-Hyg clones. Fragment lengths are indicated. **B)** Result of STR analysis of chromosomes 4, 6, 12, and 18 by capillary electrophoresis. Parental cells were homozygote for chromosome 4, 6 and 18 and heterozygote for chromosome 12. However, only the parental 67NR-Hyg chr. 12 allele was present in mBMDC/67NR-Hyg clones.
