# Supplementary material for: Co-cultivation of murine BMDCs with 67NR mouse mammary carcinoma cells give rise to highly drug resistant cells
Source: Cancer Cell Int. 2011 Jun 28;11:21. doi: 10.1186/1475-2867-11-21 (PMC3135493; doi:10.1186/1475-2867-11-21)
Supplement: Additional file 2 — SNP analysis of mBMDC/67NR-Hyg clones. Primer sequences appear in blue. Known polymorphisms are shown in red, whereas unknown polymorphisms are marked in green. SNPs located on chromosome 1, 3, 5, 11, 13 and 16 were analyzed. However, only the parental 67NR-Hyg SNPs were present in mBMDC/67NR-Hyg clones. [file 1475-2867-11-21-S2.DOC]

**Additional File 2**

**Chromosome 1 – marker rs32800995**

**rs32800995** CATGGACCAATGCTTAGAAGGGAGTCAGCTCTG**W**TGTGCACCATGGAGAA **50**

Chr1-67NR-Hyg-Chr1-rev CATGGACCAATGCTTAGAAGGGAGTCAGCTCTG**A**TGTGCACCATGGAGAA **50**

Chr1-Klon1-Chr1-rev CATGGACCAATGCTTAGAAGGGAGTCAGCTCTG**A**TGTGCACCATGGAGAA **50**

Chr1-Klon2-Chr1-rev CATGGACCAATGCTTAGAAGGGAGTCAGCTCTG**A**TGTGCACCATGGAGAA **50**

Chr1-Klon3-Chr1-rev CATGGACCAATGCTTAGAAGGGAGTCAGCTCTG**A**TGTGCACCATGGAGAA **50**

Chr1-GFP-Niere-Chr1-rev CATGGACCAATGCTTAGAAGGGAGTCAGCTCTG**T**TGTGCACCATGGAGAA **50**

********************************* ****************

**rs32800995** TATAATTCCCCCAAATCCTCGCATCTGGATCTTGATTTTTATATCGACAT **100**

Chr1-67NR-Hyg-Chr1-rev TATAATTCCCCCAAATCCTCGCATCTGGATCTTGATTTTTATATCGACAT **100**

Chr1-Klon1-Chr1-rev TATAATTCCCCCAAATCCTCGCATCTGGATCTTGATTTTTATATCGACAT **100**

Chr1-Klon2-Chr1-rev TATAATTCCCCCAAATCCTCGCATCTGGATCTTGATTTTTATATCGACAT **100**

Chr1-Klon3-Chr1-rev TATAATTCCCCCAAATCCTCGCATCTGGATCTTGATTTTTATATCGACAT **100**

Chr1-GFP-Niere-Chr1-rev TATAATTCCCCCAAATCCTCGCATCTGGATCTTGATTTTTATATCGACAT **100**

**************************************************

**rs32800995** AGGAGCATATGTCGACTGGGCTCTGGAGAAACAACAAGGCATTGTTGCCC **150**

Chr1-67NR-Hyg-Chr1-rev AGGAGCATATGTCGACTGGGCTCTGGAGAAACAACAAGGCATTGTTGCCC **150**

Chr1-Klon1-Chr1-rev AGGAGCATATGTCGACTGGGCTCTGGAGAAACAACAAGGCATTGTTGCCC **150**

Chr1-Klon2-Chr1-rev AGGAGCATATGTCGACTGGGCTCTGGAGAAACAACAAGGCATTGTTGCCC **150**

Chr1-Klon3-Chr1-rev AGGAGCATATGTCGACTGGGCTCTGGAGAAACAACAAGGCATTGTTGCCC **150**

Chr1-GFP-Niere-Chr1-rev AGGAGCA------------------------------------------- **107**(Del 57bp)

*******

**rs32800995** TTTCCCTTTTAAAAGGGAGATTCTTTTAGTTGAAGTTAGTTGGTTACAAA **200**

Chr1-67NR-Hyg-Chr1-rev TTTCCCTTTTAAAAGGGAGATTCTTTTAGTTGAAGTTAGTTGGTTACAAA **200**

Chr1-Klon1-Chr1-rev TTTCCCTTTTAAAAGGGAGATTCTTTTAGTTGAAGTTAGTTGGTTACAAA **200**

Chr1-Klon2-Chr1-rev TTTCCCTTTTAAAAGGGAGATTCTTTTAGTTGAAGTTAGTTGGTTACAAA **200**

Chr1-Klon3-Chr1-rev TTTCCCTTTTAAAAGGGAGATTCTTTTAGTTGAAGTTAGTTGGTTACAAA **200**

Chr1-GFP-Niere-Chr1-rev --------------GGGAGATTCTTTTAGTTGAAGTTAGTTGGTTACAAA **143**

************************************

**rs32800995** G**R**TGGTGGCAC**W**TC**R**CATGGGTGGGTTGGTTGGACCTGACTCTATAGACT **250**

Chr1-67NR-Hyg-Chr1-rev G**A**TGGTGGCAC**A**TC**A**CATGGGTGGGTTGGTTGGACCTGACTCTATAGACT **250**

Chr1-Klon1-Chr1-rev G**A**TGGTGGCAC**A**TC**A**CATGGGTGGGTTGGTTGGACCTGACTCTATAGACT **250**

Chr1-Klon2-Chr1-rev G**A**TGGTGGCAC**A**TC**A**CATGGGTGGGTTGGTTGGACCTGACTCTATAGACT **250**

Chr1-Klon3-Chr1-rev G**A**TGGTGGCAC**A**TC**A**CATGGGTGGGTTGGTTGGACCTGACTCTATAGACT **250**

Chr1-GFP-Niere-Chr1-rev G**G**TGGTGGCAC**T**TC**G**CATGGGTGGGTTGGTTGGACCTGACTCTATAGACT **193**

* ********* ** ***********************************

**rs32800995** TCCACCAGTCAGGAATAGTTGACTATTTTAAGGAAGCCCTGGGAGTGAGC **300**

Chr1-67NR-Hyg-Chr1-rev TCCACCAGTCAGGAATAGTTGACTATTTTAAGGAAGCCCTGGGAGTGAGC **300**

Chr1-Klon1-Chr1-rev TCCACCAGTCAGGAATAGTTGACTATTTTAAGGAAGCCCTGGGAGTGAGC **300**

Chr1-Klon2-Chr1-rev TCCACCAGTCAGGAATAGTTGACTATTTTAAGGAAGCCCTGGGAGTGAGC **300**

Chr1-Klon3-Chr1-rev TCCACCAGTCAGGAATAGTTGACTATTTTAAGGAAGCCCTGGGAGTGAGC **300**

Chr1-GFP-Niere-Chr1-rev TCCACCAGTCAGGAATAGTTGACTATTTTAAGGAAGCCCTGGGAGTGAGC **243**

**************************************************

**rs32800995** CCTGCTGTGACTGTGTAGTGGTGGGAAATGATCTAGAACGTGTTTCTAAA **350**

Chr1-67NR-Hyg-Chr1-rev CCTGCTGTGACTGTGTA--------------------------------- **317**

Chr1-Klon1-Chr1-rev CCTGCTGTGA---------------------------------------- **310**

Chr1-Klon2-Chr1-rev CCTGCTGTGA---------------------------------------- **310**

Chr1-Klon3-Chr1-rev CCTGCTGTGA---------------------------------------- **310**

Chr1-GFP-Niere-Chr1-rev CCTGCTGTGACTGTGTA--------------------------------- **260**

**********

**rs32800995** GTGCCTCTGTT **361**bp

Chr1-67NR-Hyg-Chr1-rev -----------

Chr1-Klon1-Chr1-rev -----------

Chr1-Klon2-Chr1-rev -----------

Chr1-Klon3-Chr1-rev -----------

Chr1-GFP-Niere-Chr1-rev -----------

**Chromosome 3 – marker rs3022953**

rs3022953 CATGCTATGTGCAAACACATCTGTATTTTGTAGG**R**GCACCAGATACTGAG **50**

Chr3-2-67NR-Hyg-Chr3-2-rev CATGCTATGTGCAAACACATCTGTATTTTGTAGG**G**GCACCAGATACTGAG **50**

Chr3-2-Klon1-Chr3-2-rev CATGCTATGTGCAAACACATCTGTATTTTGTAGG**G**GCACCAGATACTGAG **50**

Chr3-2-Klon2-Chr3-2-rev CATGCTATGTGCAAACACATCTGTATTTTGTAGG**G**GCACCAGATACTGAG **50**

Chr3-2-Klon3-Chr3-2-rev CATGCTATGTGCAAACACATCTGTATTTTGTAGG**G**GCACCAGATACTGAG **50**

Chr3-2-GFP-Niere-Chr3-2-rev CATGCTATGTGCAAACACATCTGTATTTTGTAGG**A**GCACCAGATACTGAG **50**

********************************** ***************

rs3022953 AGCAAGGGCCATTTTTTCCCATTAAAGC**Y**GCCTTCTTCATTTTCTC**Y**GTC **100**

Chr3-2-67NR-Hyg-Chr3-2-rev AGCAAGGGCCATTTTTTCCCATTAAAGC**T**GCCTTCTTCATTTTCTC**T**GTC **100**

Chr3-2-Klon1-Chr3-2-rev AGCAAGGGCCATTTTTTCCCATTAAAGC**T**GCCTTCTTCATTTTCTC**T**GTC **100**

Chr3-2-Klon2-Chr3-2-rev AGCAAGGGCCATTTTTTCCCATTAAAGC**T**GCCTTCTTCATTTTCTC**T**GTC **100**

Chr3-2-Klon3-Chr3-2-rev AGCAAGGGCCATTTTTTCCCATTAAAGC**T**GCCTTCTTCATTTTCTC**T**GTC **100**

Chr3-2-GFP-Niere-Chr3-2-rev AGCAAGGGCCATTTTTTCCCATTAAAGC**C**GCCTTCTTCATTTTCTC**C**GTC **100**

**************************** ***************** ***

rs3022953 TTTCAATACTGCC**R**TTACCTATGTGGTATTGAGAGCATGTGAATGAGGTA **150**

Chr3-2-67NR-Hyg-Chr3-2-rev TTTCAATACTGCC**G**TTACCTATGTGGTATTGAGAGCATGTGAATGAGGTA **150**

Chr3-2-Klon1-Chr3-2-rev TTTCAATACTGCC**G**TTACCTATGTGGTATTGAGAGCATGTGAATGAGGT- **149**

Chr3-2-Klon2-Chr3-2-rev TTTCAATACTGCC**G**TTACCTATGTGGTATTGAGAGCATGTGAATGAGGTA **150**

Chr3-2-Klon3-Chr3-2-rev TTTCAATACTGCC**G**TTACCTATGTGGTATTGAGAGCATGTGAATGAGGTA **150**

Chr3-2-GFP-Niere-Chr3-2-rev TTTCAATACTGCC**A**TTACCTATGTGGTATTGAGAGCATGTGAATGAGGTA **150**

************* ***********************************

rs3022953 ACTCAAGTCTAGTCTTCTCCCCCTCCACACTGGGGGAAGCCAGCTGCAGC **200**

Chr3-2-67NR-Hyg-Chr3-2-rev ACTCAAGTCTAGTCTTCTCCCC---------------------------- **172**

Chr3-2-Klon1-Chr3-2-rev --------------------------------------------------

Chr3-2-Klon2-Chr3-2-rev ACTCAAGTCTAGTCTTCTCCCC---------------------------- **172**

Chr3-2-Klon3-Chr3-2-rev ACTCAAGTCTAGTCTTCTCCCC---------------------------- **172**

Chr3-2-GFP-Niere-Chr3-2-rev ACTCAAGTCTAGTCTTCTCCCC---------------------------- **172**

rs3022953 CCTGCATACCTTGTCAA **217**bp

Chr3-2-67NR-Hyg-Chr3-2-rev -----------------

Chr3-2-Klon1-Chr3-2-rev -----------------

Chr3-2-Klon2-Chr3-2-rev -----------------

Chr3-2-Klon3-Chr3-2-rev -----------------

Chr3-2-GFP-Niere-Chr3-2-rev -----------------

**Chromosome 5 – marker rs3023062**

**rs3023062** GCAAGCCCCAAAGAATTGTAACTTGCCC**R**GTTTCCGTCTGAGCCTTTGGA **50**

Chr5-67NR-Hyg-Chr5-rev GCAAGCCCCAAAGAATTGTAACTTGCCC**A**GTTTCCGTCTGAGCCTTTGGA **50**

Chr5-Klon1-Chr5-rev GCAAGCCCCAAAGAATTGTAACTTGCCC**A**GTTTCCGTCTGAGCCTTTGGA **50**

Chr5-Klon2-Chr5-rev GCAAGCCCCAAAGAATTGTAACTTGCCC**A**GTTTCCGTCTGAGCCTTTGGA **50**

Chr5-Klon3-Chr5-rev GCAAGCCCCAAAGAATTGTAACTTGCCC**A**GTTTCCGTCTGAGCCTTTGGA **50**

Chr5-GFP-Niere-Chr5-rev GCAAGCCCCAAAGAATTGTAACTTGCCC**G**GTTTCCGTCTGAGCCTTTGGA **50**

**************************** *********************

**rs3023062**

Chr5-67NR-Hyg-Chr5-rev TAAGAACTTAGCTGCCAACATTAACTACGTGAGGAAGAGAATCACAACCA **100**

Chr5-Klon1-Chr5-rev TAAGAACTTAGCTGCCAACATTAACTACGTGAGGAAGAGAATCACAACCA **100**

Chr5-Klon2-Chr5-rev TAAGAACTTAGCTGCCAACATTAACTACGTGAGGAAGAGAATCACAACCA **100**

Chr5-Klon3-Chr5-rev TAAGAACTTAGCTGCCAACATTAACTACGTGAGGAAGAGAATCACAACCA **100**

Chr5-GFP-Niere-Chr5-rev TAAGAACTTAGCTGCCAACATTAACTACGTGAGGAAGAGAATCACAACCA **100**

**************************************************

**rs3023062** CTGAGAAACAGGAAACTGCTGTTTCCCGCTAAGAGAAAGGACTCATTAGG **150**

Chr5-67NR-Hyg-Chr5-rev CTGAGAAACAGGAAACTGCTGTT--------------------------- **123**

Chr5-Klon1-Chr5-rev CTGAGAAACAGGAAACTGCTGTT--------------------------- **123**

Chr5-Klon2-Chr5-rev CTGAGAAACAGGAAACTGCTGTT--------------------------- **123**

Chr5-Klon3-Chr5-rev CT------------------------------------------------ **102**

Chr5-GFP-Niere-Chr5-rev CTGAGAAACAGGAAACTGCTGTT--------------------------- **123**

**

**rs3023062** TAGCAAACCCAACGTGCTGTTCTT **174**bp

Chr5-67NR-Hyg-Chr5-rev ------------------------

Chr5-Klon1-Chr5-rev ------------------------

Chr5-Klon2-Chr5-rev ------------------------

Chr5-Klon3-Chr5-rev ------------------------

Chr5-GFP-Niere-Chr5-rev ------------------------

**Chromosome 11 – marker rs3088673**

rs3088673 GCAATGGGACAGCTTTGATTTTAGGCTGGTGATAGTGATGGGCTCTCTCA **50**

Chr11-67NR-Hyg-Chr11-fwd ----------------------------------GTGATGGGCTCTCTCA **16**

Chr11-Klon1-Chr11-fwd ---------------------------------------GGGCTCTCTCA 11

Chr11-Klon2-Chr11-fwd --------------------------------------------------

Chr11-Klon3-Chr11-fwd --------------------------------------------------

Chr11-GFP-Niere-Chr11-fwd ---------------------------------------GGGCTCTCTCA **11**

rs3088673 TGCACAGAGATGGCATTCTGGTTCCCCTGTCACTACCAACCCTGCCAGCG **100**

Chr11-67NR-Hyg-Chr11-fwd TGCACAGAGATGGCATTCTGGTTCCCCTGTCACTACCAACCCTGCCAGCG **65**

Chr11-Klon1-Chr11-fwd TGCACAGAGATGGCATTCTGGTTCCCCTGTCACTACCAACCCTGCCAGCG **61**

Chr11-Klon2-Chr11-fwd TGCACAGAGATGGCATTCTGGTTCCCCTGTCACTACCAACCCTGCCAGCG **50**

Chr11-Klon3-Chr11-fwd ---------ATGGCATTCTGGTTCCCCTGTCACTACCAACCCTGCCAGCG **41**

Chr11-GFP-Niere-Chr11-fwd TGCACAGAGATGGCATTCTGGTTCCCCTGTCACTACCAACCCTGCCAGCG **61**

*****************************************

rs3088673 **M**TATGCACCCTGCTGTATCTGTCAGCCGTGCCGTCTTGTTGGGAAACTCATGGGT **155**bp

Chr11-67NR-Hyg-Chr11-fwd **C**TATGCACCCTGCTGTATCTGTCAGCCGTGCCGTCTTGTTGGGAAACTCATGGGT **136**

Chr11-Klon1-Chr11-fwd **C**TATGCACCCTGCTGTATCTGTCAGCCGTGCCGTCTTGTTGGGAAACTCATGGGT **132**

Chr11-Klon2-Chr11-fwd **C**TATGCACCCTGCTGTATCTGTCAGCCGTGCCGTCTTGTTGGGAAACTCATGGGT **121**

Chr11-Klon3-Chr11-fwd **C**TATGCACCCTGCTGTATCTGTCAGCCGTGCCGTCTTGTTGGGAAACTCATGGGT **112**

Chr11-GFP-Niere-Chr11-fwd **A**TATGCACCCTGCTGTATCTGTCAGCCGTGCCGTCTTGTTGGGAAACTCATGGGT **132**

******************************************************

**Chromosome 13 – marker rs3023382**

rs3023382 CCATGAGCCTTGAAGAAGGAGTGTCACATGGATTTGCACAGTCAGATGGA **50**

Chr13-67NR-Hyg-Chr13-fwd -----------------------------------------------GGA **3**

Chr13-Klon1-Chr13-fwd -----------------------------------------------GGA **3**

Chr13-Klon2-Chr13-fwd -----------------------------------------------GGA **3**

Chr13-Klon3-Chr13-fwd -----------------------------------------------GGA **3**

Chr13-GFP-Niere-Chr13-fwd -----------------------------------------------GGA **3**

***

rs3023382 GCCACCCAGTGTGTGCCCTAGTGTCTGCGACCTCAGGCAGA**K**CTGCTGGA **100**

Chr13-67NR-Hyg-Chr13-fwd GCCACCCAGTGTGTGCCCTAGTGTCTGCGACCTCAGGCAGA**G**CTGCTGGA **53**

Chr13-Klon1-Chr13-fwd GCCACCCAGTGTGTGCCCTAGTGTCTGCGACCTCAGGCAGA**G**CTGCTGGA **53**

Chr13-Klon2-Chr13-fwd GCCACCCAGTGTGTGCCCTAGTGTCTGCGACCTCAGGCAGA**G**CTGCTGGA **53**

Chr13-Klon3-Chr13-fwd GCCACCCAGTGTGTGCCCTAGTGTCTGCGACCTCAGGCAGA**G**CTGCTGGA **53**

Chr13-GFP-Niere-Chr13-fwd GCCACCCAGTGTGTGCCCTAGTGTCTGCGACCTCAGGCAGA**T**CTGCTGGA **53**

***************************************** ********

rs3023382 AAGGTAAGTTCTACTGCACTGAGGTTTGCACGAATTCCCCCGCCACCCGC **150**

Chr13-67NR-Hyg-Chr13-fwd AAGGTAAGTTCTACTGCACTGAGGTTTGCACGAATTCCCCCGCCACCCGC **103**

Chr13-Klon1-Chr13-fwd AAGGTAAGTTCTACTGCACTGAGGTTTGCACGAATTCCCCCGCCACCCGC **103**

Chr13-Klon2-Chr13-fwd AAGGTAAGTTCTACTGCACTGAGGTTTGCACGAATTCCCCCGCCACCCGC **103**

Chr13-Klon3-Chr13-fwd AAGGTAAGTTCTACTGCACTGAGGTTTGCACGAATTCCCCCGCCACCCGC **103**

Chr13-GFP-Niere-Chr13-fwd AAGGTAAGTTCTACTGCACTGAGGTTTGCACGAATTCCCCCGCCACCCGC **103**

**************************************************

rs3023382 CGGAGTCTCATGTGTACATGCATGCACATACTTACATACCCAT **193**bp

Chr13-67NR-Hyg-Chr13-fwd CGGAGTCTCATGTGTACATGCATGCACATACTTACATACCCAT **146**

Chr13-Klon1-Chr13-fwd CGGAGTCTCATGTGTACATGCATGCACATACTTACATACCCAT **146**

Chr13-Klon2-Chr13-fwd CGGAGTCTCATGTGTACATGCATGCACATACTTACATACCCAT **146**

Chr13-Klon3-Chr13-fwd CGGAGTCTCATGTGTACATGCATGCACATACTTACATACCCAT **146**

Chr13-GFP-Niere-Chr13-fwd CGGAGTCTCATGTGTACATGCATGCACATACTTACATACCCAT **146**

*******************************************

blue: primer sequence; red: known polymorphism; green: unknown polymorphism

**Chromosome 16 – marker rs3023435**

rs3023435 CATGGCACCAATATCGTCTCTGATCTAAATGACAGTTCATCCCCCTCTTT **50**

Chr16-67NR-Hyg-Chr16-fwd ------------------------------------------------TT **2**

Chr16-Klon1-Chr16-fwd ------------------------------------------------TT **2**

Chr16-Klon2-Chr16-fwd ------------------------------------------------TT **2**

Chr16-Klon3-Chr16-fwd ------------------------------------------------TT **2**

Chr16-GFP-Niere-Chr16-fwd ------------------------------------------------TT **2**

------------------------------------------------**

rs3023435 TCTGGTGTCTTTTCAACTCACATTTCACGTAGAAATAAATATTTTAAAAT **100**

Chr16-67NR-Hyg-Chr16-fwd TCTGGTGTCTTTTCAACTCACATTTCACGTAGAAATAAATATTTTAAAAT **52**

Chr16-Klon1-Chr16-fwd TCTGGTGTCTTTTCAACTCACATTTCACGTAGAAATAAATATTTTAAAAT **52**

Chr16-Klon2-Chr16-fwd TCTGGTGTCTTTTCAACTCACATTTCACGTAGAAATAAATATTTTAAAAT **52**

Chr16-Klon3-Chr16-fwd TCTGGTGTCTTTTCAACTCACATTTCACGTAGAAATAAATATTTTAAAAT **52**

Chr16-GFP-Niere-Chr16-fwd TCTGGTGTCTTTTCAACTCACATTTCACGTAGAAATAAATATTTTAAAAT **52**

**************************************************

rs3023435 ATAAAAACAAATATTCAGTTAAAAGAAAACCAGAGGACTT-CTTTATATT **150**

Chr16-67NR-Hyg-Chr16-fwd ATAAAAACAAATATTCAGTTAAAAGAAAACCAGAGGACTTTCTTTATATT **102**

Chr16-Klon1-Chr16-fwd ATAAAAACAAATATTCAGTTAAAAGAAAACCAGAGGACTTTCTTTATATT **102**

Chr16-Klon2-Chr16-fwd ATAAAAACAAATATTCAGTTAAAAGAAAACCAGAGGACTTTCTTTATATT **102**

Chr16-Klon3-Chr16-fwd ATAAAAACAAATATTCAGTTAAAAGAAAACCAGAGGACTTTCTTTATATT **102**

Chr16-GFP-Niere-Chr16-fwd ATAAAAACAAATATTCAGTTAAAAGAAAACCAGAGGACTTTCTTTATATT **102**

**************************************** *********

rs3023435 -AGATAAAAC**R**GACAGGAAAATCCTACAGGTATTCATCAGCAATGTGACA **200**

Chr16-67NR-Hyg-Chr16-fwd CAGATAAAAC**G**GACAGGAAAATCCTACAGGTATTCATCAGCAATGTGACA **152**

Chr16-Klon1-Chr16-fwd CAGATAAAAC**G**GACAGGAAAATCCTACAGGTATTCATCAGCAATGTGACA **152**

Chr16-Klon2-Chr16-fwd CAGATAAAAC**G**GACAGGAAAATCCTACAGGTATTCATCAGCAATGTGACA **152**

Chr16-Klon3-Chr16-fwd CAGATAAAAC**G**GACAGGAAAATCCTACAGGTATTCATCAGCAATGTGACA **152**

Chr16-GFP-Niere-Chr16-fwd CAGATAAAAC**A**GACAGGAAAATCCTACAGGTATTCATCAGCAATGTGACA **152**

********* ***************************************

rs3023435 ACCCCATGAAGCAACAGTGTCAACTATTTCTGCCACTCTTCCCTTGTCCA **250**

Chr16-67NR-Hyg-Chr16-fwd ACCCCATGAAGCAACAGTGTCAACTATTTCTGCCACTCTTCCCTTGTCCA **202**

Chr16-Klon1-Chr16-fwd ACCCCATGAAGCAACAGTGTCAACTATTTCTGCCACTCTTCCCTTGTCCA **202**

Chr16-Klon2-Chr16-fwd ACCCCATGAAGCAACAGTGTCAACTATTTCTGCCACTCTTCCCTTGTCCA **202**

Chr16-Klon3-Chr16-fwd ACCCCATGAAGCAACAGTGTCAACTATTTCTGCCACTCTTCCCTTGTCCA **202**

Chr16-GFP-Niere-Chr16-fwd ACCCCATGAAGCAACAGTGTCAACTATTTCTGCCACTCTTCCCTTGTCCA **202**

**************************************************

rs3023435 TAATGAATGCT-T**Y**GGGACACTGGCCTGACTGACTCAGCTCCAAGGACTT **300**

Chr16-67NR-Hyg-Chr16-fwd TAATGAATGCTCT**T**GGGACACTGGCCTGACTGACTCAGCTCCAAGGACTT **252**

Chr16-Klon1-Chr16-fwd TAATGAATGCTCT**T**GGGACACTGGCCTGACTGACTCAGCTCCAAGGACTT **252**

Chr16-Klon2-Chr16-fwd TAATGAATGCTCT**T**GGGACACTGGCCTGACTGACTCAGCTCCAAGGACTT **252**

Chr16-Klon3-Chr16-fwd TAATGAATGCTCT**T**GGGACACTGGCCTGACTGACTCAGCTCCAAGGACTT **252**

Chr16-GFP-Niere-Chr16-fwd TAATGAATGCTCT**C**GGGACACTGGCCTGACTGACTCAGCTCCAAGGACTT **252**

************* ************************************

rs3023435 TCCTATCTTGAAAGGATTGCT **321**bp

Chr16-67NR-Hyg-Chr16-fwd TCCTATCTTGAAAGGATTGCT **273**

Chr16-67NR-Hyg-Chr16-fwd TCCTATCTTGAAAGGATTGCT **273**

Chr16-Klon1-Chr16-fwd TCCTATCTTGAAAGGATTGCT **273**

Chr16-Klon2-Chr16-fwd TCCTATCTTGAAAGGATTGCT **273**

Chr16-Klon3-Chr16-fwd TCCTATCTTGAAAGGATTGCT **273**

Chr16-GFP-Niere-Chr16-fwd TCCTATCTTGAAAGGATTGCT **273**

*********************

**SNP analysis of mBMDC/67NR-Hyg clones.** Primer sequences appear in blue. Known polymorphisms are shown in red, whereas unknown polymorphisms are marked in green. SNPs located on chromosome 1, 3, 5, 11, 13 and 16 were analyzed. However, only the parental 67NR-Hyg SNPs were present in mBMDC/67NR-Hyg clones.
