# Supplementary material for: Co-cultivation of murine BMDCs with 67NR mouse mammary carcinoma cells give rise to highly drug resistant cells
Source: Cancer Cell Int. 2011 Jun 28;11:21. doi: 10.1186/1475-2867-11-21 (PMC3135493; doi:10.1186/1475-2867-11-21)
Supplement: Additional file 3 — Summary of primer pairs for STR analysis [file 1475-2867-11-21-S3.DOC]

**Additional file 3: Summary of primer pairs for STR analysis**

| Name | MGI Accession ID | Mean product size | Primer | Sequence (5’ to 3’) |
| --- | --- | --- | --- | --- |
| Chr. 4 | MGI: | 174bp† | forward | ACTGAATCTCCGCGAGGAAAGCGAACT |
|  | 1340218 | 129bp* | reverse | GAATCGGGGTACGACCGAAAGAGT |
| Chr. 6 | MGI: | 195bp† | forward | CAAGCTGCCTTTGCACATGGC |
|  | 224 | 180bp* | reverse | CCATGGGTCCATAGCTCGGGC |
| Chr. 12 | MGI: | 332bp† | forward | ATAGGACGGGGAGAACGCCTC |
|  | 393 | 334bp* | reverse | GGCAAGTTCATCAGCTAGTGC |
| Chr. 17 | MGI: | 108bp† | forward | GGCTTCCACACATGATTGC |
|  | 703211 | 125bp* | reverse | TTCTGGGTCCATCATCACAA |
| Chr. 18 | MGI: | 176bp† | forward | ATGTGTATTTGTGTATATGT |
|  | 1890109 | 178bp* | reverse | CCCTTAGATAGGACAGAACTG |

† 67NR-Hyg (host strain Balb/c)

* BMDC (Tg/ACTB-EGFP)B5Nagy/J (003115): host strain 129S1/Sv × 129X1/SvJ))
