# Supplementary material for: Co-cultivation of murine BMDCs with 67NR mouse mammary carcinoma cells give rise to highly drug resistant cells
Source: Cancer Cell Int. 2011 Jun 28;11:21. doi: 10.1186/1475-2867-11-21 (PMC3135493; doi:10.1186/1475-2867-11-21)
Supplement: Additional file 4 — Summary of primer pairs for SNP analysis [file 1475-2867-11-21-S4.DOC]

**Additional file 4: Summary of primer pairs for SNP analysis**

| Name | Marker | SNP | Primer | Sequence (5’ to 3’) |
| --- | --- | --- | --- | --- |
| Chr. 1 | rs3280995 | A† | forward | CATGGACCAATGCTTAGAAG |
|  |  | T* | reverse | AACAGAGGCACTTTAGAAACA |
| Chr. 3 | rs3022953 | T† | forward | CATGCTATGTGCAAACACATC |
|  |  | C* | reverse | TTGACAAGGTATGCAGGGCT |
| Chr. 5 | rs3023062 | A† | forward | GCAAGCCCCAAAGAATTGTA |
|  |  | G* | reverse | AAGAACAGCACGTTGGGTTT |
| Chr. 11 | rs3088673 | C† | forward | GCAATGGGACAGCTTTGATT |
|  |  | A* | reverse | ACCCATGAGTTTCCCAACAA |
| Chr. 13 | rs3023382 | G† | forward | CCATGAGCCTTGAAGAAGGA |
|  |  | T* | reverse | ATGGGTATGTAAGTATGTGC |
| Chr. 16 | rs3023435 | T† | forward | CATGGAACCAATATCGTCTC |
|  |  | C* | reverse | CAGCAATCCTTTCAAGATAGG |

† 67NR-Hyg (host strain Balb/c)

* BMDC (Tg/ACTB-EGFP)B5Nagy/J (003115))
